# Supplementary material for: Signal Peptide Variants in Inherited Retinal Diseases: A Multi-Institutional Case Series
Source: Int J Mol Sci. 2022 Nov 1;23(21):13361. doi: 10.3390/ijms232113361 (PMC9658040; doi:10.3390/ijms232113361)
Supplement: Supplementary file 1 [file ijms-23-13361-s001.zip › ijms-1931656-supplementary.pdf]

Table S1. Genes and mapped loci causing retinal diseases that possess signal peptide sequences and their corresponding chromosomal location, length, and position.*

Gene Symbol	Mapped Loci	SP Length	SP Position
**Chromosome 1**			
EMC1	1p36.13	22	1-22
PLA2G5	1p36.13-p36.12	20	1-20
COL11A1	1p21.1	35	1-35
CLCC1	1p13.3	18	1-18
SEMA4A	1q22	32	1-32
HMCN1	1q25.3-q31.1	21	1-21
CFH	1q31.3	18	1-18
CRB1	1q31.3	25	1-25
USH2A	1q41	31	1-31
**Chromosome 2**			
EFEMP1	2p16.1	17	1-17
MERTK	2q13	20	1-20
SPP2	2q37.1	29	1-29
**Chromosome 3**			
IMPG2	3q12.3	22	1-22
**Chromosome 4**			
PROM1	4p15.32	19	1-19
ADGRA3	4p15.2	33	1-33
MTTP	4q23	18	1-18
LRIT3	4q25	19	1-19
TLR3	4q35.1	23	1-23
**Chromosome 5**			
MCDR3$^{\pm}$	5p15.33-p13.1	20	1-20
ADGRV1	5q14.3	29	1-29
GRM6	5q35.3	24	1-24

**Chromosome 6**			
C2	6p21.32	20	1-20
CFB	6p21.32	25	1-25
EYS	6q12	21	1-21
COL9A1	6q13	23	1-23
IMPG1	6q14.1	20	1-20
**Chromosome 8**			
ADAM9	8p11.23	28	1-28
GDF6	8q22.1	22	1-22
**Chromosome 9**			
TLR4	9q33.1	23	1-23
**Chromosome 10**			
RBP3	10q11.22	17	1-17
PCDH15	10q21.1	26	1-26
CDH23	10q22.1	23	1-23
CDHR1	10q23.1	19	1-19
RBP4	10q23.33	18	1-18
HTRA1	10q26.13	22	1-22
**Chromosome 11**			
FZD4	11q14.2	36	1-36
LRP5		31	1-31
C1QTNF5	11q23.3	15	1-37
**Chromosome 12**			
CACNA2D4	12p13.33	19	1-19
COL2A1	12q13.11	25	1-25
**Chromosome 14**			
FBLN5	14q32.12	23	1-23
**Chromosome 15**			
SLC24A1	15q22.31	NOT CLEAVED	1 - ?

**Chromosome 16**			
GNPTG	16p13.3	24	1-24
CDH3	16q22.1	24	1-24
ADAMTS18	16q23.1	47	1-47
**Chromosome 17**			
GUCY2D	17p13.1	51	1-51
GPR179	17q12	25	1-25
CA4	17q23.2	18	1-18
ARSG	17q24.2	16	1-16
**Chromosome 18**			
LAMA1	18p11.31-p11.23	17	1-17
**Chromosome 19**			
C3	19p13.3	22	1-22
**Chromosome 20**			
JAG1	20p12.2	33	1-33
**Chromosome 22**			
TIMP3	22q12.3	23	1-23
**Chromosome Y**			
RS1	Xp22.13	23	1-23
NYX	Xp11.4	23	1-23
NDP	Xp11.3	24	1-24

*Obtained from https://www.uniprot.org/

± ***Corresponds to genetic locus***
